# Risk Factors and Outcomes in Dogs With Respiratory Disease Undergoing Diagnostic Airway Lavage

**DOI:** 10.3389/fvets.2020.00165

**Published:** 2020-04-17

**Authors:** Zoe Bianco, Alex Bukoski, Isabelle Masseau, Colin Reich, Loren Schultz, Carol Reinero

**Affiliations:** ^1^Department of Veterinary Medicine and Surgery, Veterinary Health Center, University of Missouri, Columbia, MO, United States; ^2^Department of Clinical Sciences, Faculté de Médecine Vétérinaire, Université de Montréal, St. Hyacinthe, QC, Canada

**Keywords:** bronchoalveolar lavage, thoracic computed tomography, disease severity scoring, pulmonary parenchymal disease, airway disorders, pulmonary vascular disease, ventilator-acquired pulmonary mechanics

## Abstract

Advanced diagnostic testing is becoming increasingly important to accurately assess pulmonary parenchymal, airway, and pulmonary vascular diseases in dogs. Due to respiratory system compromise, diagnostic procedures performed under general anesthesia, including thoracic computed tomography (CT) and bronchoalveolar lavage (BAL), are thought to carry significant risk to dogs with respiratory disease. In lieu of performing these diagnostics, empirical medical therapy is often administered, potentially delaying appropriate therapy or providing unnecessary treatment. This study prospectively evaluated risk factors and outcomes for dogs with respiratory disease undergoing general anesthesia for thoracic CT and BAL. Arterial blood gas samples were taken pre- and post-BAL to evaluate pulmonary gas exchange. Pre-BAL arterial partial pressure of oxygen-to-fractional inspired oxygen ratio was used to stratify dogs into groups of mild or moderate to severe disease severity. A novel thoracic CT disease severity scoring system was used to independently stratify dogs into mild or moderate to severe groups. Statistical comparisons between groups were made for signalment, body weight, temperature, pulse, respiratory rate, WBC count, ventilator-acquired pulmonary mechanics (specific compliance and resistance), change in arterial partial pressure of oxygen post-BAL, and outcomes. Seventeen dogs were prospectively enrolled. A comparatively lower heart rate at presentation was the only potential marker of increased disease severity identified when stratified by CT severity score. Arterial partial pressure of oxygen did not significantly decrease post-BAL regardless of disease severity or stratification method. The CT scoring system significantly correlated with the pre-BAL arterial partial pressure of oxygen-to-fractional inspired oxygen ratio. Incidence of post-procedural complications was 18%, with all complications being transient. Mortality as a direct complication of diagnostics was 0%. When considering euthanasia secondary to severity of the underlying disease and poor prognosis or death due to unrelated disease, mortality was 18%. In dogs with respiratory disease undergoing advanced diagnostic procedures, the overall incidence of post-procedural morbidity was low with no mortality directly attributed to the procedures. A novel CT disease severity scoring system was utilized and shows promise as a tool for evaluation of disease severity in this patient population when compared to arterial blood gas analysis.

## Introduction

Advanced diagnostic testing is becoming increasingly important to accurately assess pulmonary parenchymal, airway, and pulmonary vascular diseases in dogs. Thoracic computed tomography (CT) with inspiratory and expiratory breath-holds and angiography, tracheobronchoscopy, and collection of bronchoalveolar lavage (BAL) require general anesthesia (GA) but can provide additional diagnostic and prognostic information beyond that of traditional thoracic radiography. With increasing severity and complexity of illness, such as that seen with acute respiratory distress syndrome (ARDS), antimicrobial-resistant and recurrent bacterial pneumonia, interstitial lung disease, bronchomalacia, bronchiolar disorders, pulmonary veno-occlusive disease, and thromboembolic disease, the benefit of advanced airway diagnostics may increase.

GA and BAL are perceived to carry significant risk to dogs with respiratory disease (RD) due to the potential for direct depression of respiratory drive, compromise of cardiovascular function, and worsening of preexisting ventilation and perfusion mismatching. Unfortunately, the population of dogs that may stand to gain the greatest benefit from advanced airway diagnostics commonly have the greatest pulmonary function compromise. Therefore, due to the perceived risks of advanced diagnostic testing, empirical medical therapy is often elected instead. An objective assessment of the possible peri-procedural complications associated with these advanced diagnostic tests in dogs with RD does not currently exist in the veterinary literature. As a result, this perception of risk may delay accurate diagnosis and optimal treatment and promote inappropriate empirical antimicrobial therapy or other unnecessary treatment in this patient population.

To date, the veterinary literature addressing advanced diagnostic testing for RD is focused primarily on evaluating the diagnostic utility of thoracic CT and BAL rather than the complications associated with them. Most publications do not present data on post-procedural complications or mortality, so conclusions regarding risk are primarily anecdotal. Reported post-procedural complications in dogs undergoing BAL include the need for supplemental oxygen, bronchospasm, and worsening of preexisting RD or cough (1). A single clinical report exists that describes severe bronchoconstriction immediately following BAL in a dog with eosinophilic airway disease that also required mechanical positive pressure ventilation (PPV) (2). A retrospective study including 101 dogs undergoing BAL reported a 2% mortality, in which the two dogs that died had severe systemic disease (sepsis and metastatic cancer) prior to diagnostic testing (3). These data suggest that the overall incidence of clinically significant post-procedural complications and mortality associated with BAL is low and that disease severity and etiology may play a role in outcome.

The role of disease severity is unclear when considering the risk for dogs undergoing thoracic CT and BAL. Objectively evaluating disease severity in this patient population is challenging due to the diversity in underlying disease etiology and variations in anatomic location of disease. For example, a dog with diffuse pulmonary parenchymal disease may be at higher risk for adverse events following BAL than one with diffuse inflammatory lower airway disease, even if both are considered severely affected. Therefore, an objective measure of disease severity is needed to better assess risk in this patient population. The arterial partial pressure of oxygen-to-fractional inspired oxygen ratio (P:F) quantifies the efficiency of pulmonary gas exchange. It is commonly used in human studies to quantify disease severity in patients with pulmonary parenchymal disease (4). Although no current veterinary data exist evaluating outcomes of BAL in dogs using P:F, it has been identified as an indicator of disease severity and outcomes in dogs with ARDS, where a P:F of ≤ 300 mm Hg is a component of the diagnostic criteria for the syndrome (5). A decreasing P:F serves as an objective measure of decreased pulmonary gas exchange capability and therefore reflects increasing disease severity. Other objective metrics, including pulmonary mechanics (specific compliance and resistance), could also be used to evaluate the severity of pulmonary parenchymal disease in these dogs. However, P:F and pulmonary mechanics data are not routinely performed as they require invasive blood sampling and GA/mechanical ventilation, respectively. Alternatively, thoracic CT is a commonly recommended advanced diagnostic test for this patient population at our institution and others and may allow for quantification of disease severity without additional laboratory tests or invasive measures.

As compared to traditional thoracic radiography, thoracic CT with inspiratory and expiratory breath holds and angiography provides superior data to describe pulmonary parenchymal, airway, and pulmonary vascular diseases (6). It may also provide a more holistic and sensitive representation of disease severity than the P:F or pulmonary mechanics. In the past, thoracic CT disease scoring systems have been found to be associated with disease severity and outcomes in people with asthma, idiopathic and cystic pulmonary fibrosis, and sarcoidosis (7–11). A veterinary CT disease severity scoring system was developed for dogs with *Angiostrongylus vasorum* to describe distribution and severity of pulmonary parenchymal attenuation, but the relationship between disease severity and outcomes was not assessed (12). A more comprehensive veterinary CT disease severity scoring system that accounts for disease beyond the pulmonary parenchyma is needed to objectively and holistically quantify RD severity.

In order to fill knowledge gaps in the veterinary literature regarding procedural complications in dogs undergoing advanced diagnostic testing for RD, a prospective study was designed. Our primary objectives were to describe outcomes for dogs undergoing advanced diagnostics for RD and to evaluate the effect of BAL on pulmonary gas exchange in dogs stratified by disease severity. Secondary objectives included evaluating the utility of minimally invasive markers [Systemic Inflammatory Response Syndrome (SIRS) criteria, which include temperature, pulse, respiratory rate and WBC count], P:F, pulmonary mechanics, and a novel thoracic CT disease severity scoring system to quantify disease severity in this patient population. We hypothesized that dogs undergoing advanced diagnostic testing for RD would have a low incidence of post-procedural complications and mortality and that disease severity would be significantly associated with outcomes.

## Materials and Methods

### Study Design and Animals

A prospective, observational, single-center study was conducted between April 2018 and August 2019. The study protocol was approved by the University of Missouri Animal Care and Use Committee (IACUC #9286) and informed client consent was obtained prior to study enrollment. Client owned dogs ≥5 kg who presented to the University of Missouri Veterinary Health Center with clinical signs localizing to the respiratory tract and undergoing respiratory diagnostics were screened for enrollment. Study inclusion required arterial blood gas (ABG) analysis, thoracic CT (Aquilion 64, Canon Medical, Tustin CA) using a critical care ventilator (Engström Carestation, GE Healthcare, Finland) for paired inspiratory/expiratory breath holds and compliance and resistance measurements, and BAL. Dogs were excluded if they had evidence of uncontrolled congestive heart failure or had clinical signs localized only to the upper airway (e.g., stridor, inspiratory respiratory distress). Data recorded for each study participant at time of enrollment included signalment, body weight (kg), body condition score (BCS), respiratory rate, heart rate, and rectal body temperature. The decision to perform a WBC count was left to the discretion of the attending clinician and so was included if obtained within 1 week of presentation, as long as no clinical decompensation was noted between the time of WBC count and BAL.

### Diagnostic Procedures

Dogs had individual protocols for GA developed by a board-certified veterinary anesthesiologist to facilitate advanced diagnostic testing. All dogs were induced under GA in a room housing the CT scanner using propofol (Diprivan®, Fresenius Kabi USA, LLC, Lake Zurich IL) IV titrated to effect. Anesthesia was maintained with a constant rate infusion of propofol at 0.2–0.4 mg/kg/min, given to effect. To aid in ABG collection, an arterial catheter (24-22G) was placed percutaneously in the dorsal pedal artery following premedication or while under GA. If unsuccessful, femoral artery puncture was performed by the first author with the patient under GA.

Following intubation, patients received PPV using a critical care ventilator in sternal recumbency and volume-control mode with the following initial settings: 0.4 fraction inspired oxygen (F_I_O_2_), tidal volume 10 ml/kg, respiratory rate at 10 breaths per minute, inspiratory:expiratory ratio of 1:3, and positive end expiratory pressure (PEEP) of 5 cm H_2_O (13, 14). Ventilator settings (respiratory rate, tidal volume, and/or F_I_O_2_) were adjusted if necessary to meet patient needs. CT scan acquisition was performed using inspiratory and expiratory breath holds with the expiratory breath hold having PEEP set to 0 cm H_2_O (13, 14). An additional inspiratory breath hold series was performed following administration of IV iohexol (Omnipaque®, GE Healthcare Inc., Marlborough, MA).

Using the EView software package (GE Healthcare Inc., Marlborough, MA) installed on the mechanical ventilator, dynamic compliance and resistance were computed on a breath-by-breath basis. For each patient, these values were used to compute an average dynamic compliance and resistance over the entire mechanical ventilation period. These averages were then converted to specific compliance [average compliance/weight (kg)] and resistance [average resistance/weight (kg)] to control for patient size (15).

Following CT, patients were transferred to a gas anesthetic machine and moved to the endoscopy room while maintained on 100% oxygen and allowing spontaneous respiration. Manual PPV was performed as needed to maintain peripheral oxygen saturation (SpO_2_) ≥ 93% and end-tidal carbon dioxide (EtCO_2_) 35–50 mm Hg, if possible. After at least 10 min on 100% oxygen, an ABG sample was obtained in a heparinized arterial blood sampler (AirLife^TM^, Carefusion, Vernon Hills, IL). EtCO_2_ and body temperature were recorded at the time of collection. Samples were immediately placed on ice and analyzed within 30 min. A single point of care blood gas machine (StatProfile® PRIME™ CCS Analyzer, NOVA® Biomedical Corporation, Waltham MA) was used for all analyses.

After initial ABG collection, BAL and, if applicable, tracheobronchoscopic examination were performed. To facilitate these procedures, patients were extubated and insufflation of 100% oxygen at 1–2 L/min was provided via a red rubber catheter placed in the trachea. Airway lavage was performed as previously described (16) using up to two 20-ml aliquots of warmed sterile saline. Lavage samples were submitted for cytology (cellular differential and microscopic description) and culture (aerobic and anaerobic with sensitivity).

Following BAL, patients were reintubated and maintained on 100% oxygen for at least 10 min before a second ABG was obtained. Patients were then weaned from GA, extubated and provided supplemental oxygen via oxygen cage, nasal cannulas, and/or an oxygen mask or hood if indicated. If unable to oxygenate and/or ventilate adequately with spontaneous respiration and oxygen supplementation, patients were transitioned to mechanical PPV. Supportive care, medical therapy, and discharge following the procedure were left to the discretion of the attending clinician.

### Outcomes Assessment

Outcomes compared between groups included post-procedural complications, length of hospitalization (LOH) following BAL, and survival to discharge. Post-procedural complications were defined as mild if patients that were not already oxygen dependent prior to diagnostic testing experienced tachypnea and/or increased respiratory effort that persisted despite adequate control of extrapulmonary factors (e.g., stress, pain, anxiety) and/or there was a perceived need for oxygen supplementation beyond the immediate anesthetic recovery period but within 24 h. Post-procedural complications were defined as moderate to severe if patients experienced worsening of SpO_2_ beyond the immediate anesthetic recovery period in dogs that were oxygen- or mechanical-ventilation-dependent prior to diagnostic testing and despite adequate control of extrapulmonary factors. For dogs that were oxygen-dependent or required mechanical ventilation prior to diagnostic testing, remaining oxygen-dependent or on a mechanical ventilator without worsening of pre-diagnostic SpO2 values was not considered a post-procedural complication but a reflection of the severity of underlying disease.

Length of hospitalization was counted as days in the intensive care unit following advanced diagnostic procedures. If the patient was discharged on the same day as the procedure, LOH was counted as zero days. If admitted to the hospital following the procedure, a day of hospitalization was counted for every successive 24-h period. Patients who died or were euthanized on the same day as the procedure were not included in LOH assessment.

Survival to discharge was considered either successful or failed and this outcome was measured within the hospitalization under which the data set was collected. Failure included both cardiopulmonary arrest with or without attempted cardiopulmonary resuscitation, and humane euthanasia, whether for medical, quality of life, or fiscal considerations.

### Group Stratification for Disease Severity Assessment

Following data collection, patients were stratified into two groups based on the pre-BAL P:F. Normal P:F in dogs is >400, while values <300 and <200 are considered moderately to severely hypoxemic, respectively (5). Therefore, dogs with a P:F >300 were stratified to ABG group 1 (normoxemia or mild hypoxemia) and ≤ 300 to ABG group 2 (moderate to severe hypoxemia).

**Figure 1:**
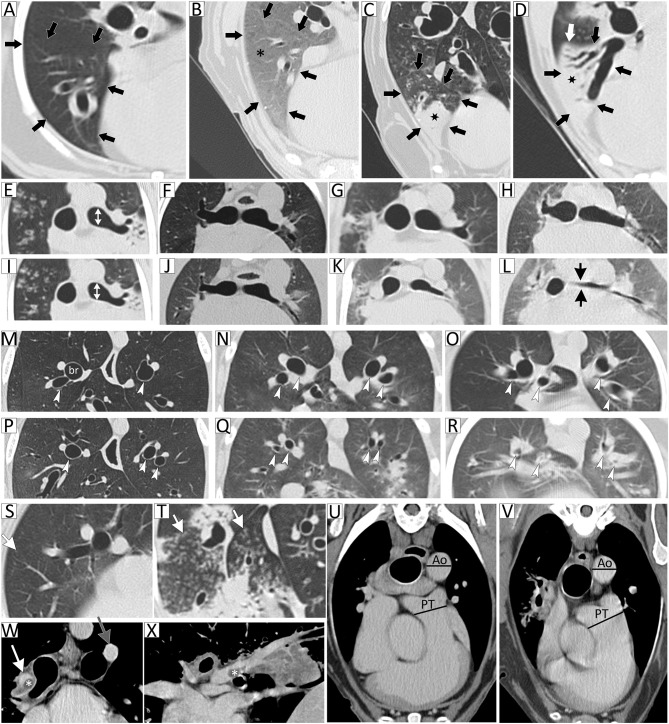
Thoracic computed tomography (CT) disease severity score categorical examples. Lung attenuation category represented by images **(A–D)** of the right middle lung lobe (outlined by black arrows). **(A)** Normal parenchymal attenuation, **(B)** primarily ground-glass opacity (GGO; asterisk), **(C)** GGO with consolidation (marked by star), and **(D)** predominant consolidation. In this example, the entire lung lobe is consolidated and the lung margin is visualized (white arrow). Airway caliber changes category, subcategory tracheal or principal bronchi narrowing, is represented by images **(E–L)**. Images are paired inspiratory **(E–H)** and expiratory **(I–L)** series. **(E,I)** are normal with white double-headed arrows indicating diameter of principal bronchus, **(F,J)** <1/3 diameter reduction of principal bronchial caliber comparing inspiratory to expiratory (I/E) series, **(G,K)** ≥1/3 to 2/3 diameter reduction of principal bronchial caliber on I/E series, and **(H,L)** ≥2/3 diameter reduction of principal bronchial caliber on I/E series. Airway caliber changes category, subcategory bronchomalacia, is represented by images **(M–R)**. Images are paired inspiratory **(M–O)** and expiratory **(P–R)** series. **(M,P)** are normal with white arrowheads indicating normal bronchi, **(N,Q)** highlight the dynamic expiratory distorted circular appearance to bronchi with moderate peribronchovascular opacification (PBVO), and **(O,R)** represent dynamic near-complete disappearance of airway lumen with marked PBVO on expiration. Airway caliber changes category, subcategory tree-in-bud, is represented by images **(S,T)**, **(S)** showing occasional tree-in-bud (arrow) and **(T)** severely affected. Vascular disease category, subcategory pulmonary thromboembolism, is represented by images **(W,X)**, showing an intraluminal filling defect consistent with a thrombus (marked by asterisk) in the right main pulmonary artery and extending into lobar artery on transverse **(W)** and sagittal **(X)** views. Vascular disease category, subcategory pulmonary trunk to descending aorta ratio (PT:Ao), is represented by images **(U,V)**, with **(U)** being normal and **(V)** with PT:Ao >1.4 suggestive of pulmonary hypertension.

Patients were also independently stratified into groups using thoracic CT disease severity scores. Thoracic CT images were interpreted and scored by a single board-certified veterinary radiologist blinded to clinical disease severity and suspected diagnoses. A CT disease severity scoring system (Table 1) was developed to independently evaluate the following three categories: (1) volume and magnitude of pulmonary parenchymal attenuation predominantly reflective of interstitial and alveolar disease; (2) airway narrowing and/or obstruction indicative of large and small airway disease, respectively; and (3) vascular changes reflective of pulmonary hypertension or vascular obstruction. The maximal score for each category (parenchymal, airway, and vasculature) was three and the total disease severity score maximum was nine, with increasing scores reflecting increasing respiratory tract involvement and disease severity. See Figure 1 for examples of CT images representative of each category. Since any patient who scored 3 in any single category was considered to have clinically impactful disease, a patient with a total CT score <3 was stratified to CT group 1 (mild disease) and a patient with a total CT score ≥3 was stratified to CT group 2 (moderate to severe disease).

**Table 1:** Thoracic computed tomography (CT) disease severity scoring system in the dog, encompassing global changes reflective of pulmonary parenchymal disease, airway disorders, and pulmonary vascular disease.

**Category**		**Sub-score**	**Final score**
Attenuation	Volume	0.5 = <1/3 of lung volume affected 1.0 = ≥1/3 to <2/3 of lung volume affected 1.5 = ≥2/3 of lung volume affected	The sum of the averaged scores for volume and opacification for each lung lobe, maximum score of 3
	Opacification	0.5 = GGO (6) 1.0 = GGO and consolidation 1.5 = Consolidation predominant	
Airway Caliber Changes	Tracheal or principal bronchial I/E narrowing	1.0 = <1/3 diameter reduction 2.0 = ≥1/3 to <2/3 diameter reduction 3.0 = ≥2/3 diameter reduction	Highest score from any category, maximum score of 3
	Bronchomalacia	1.0 = Subtle flattening with no PBVO (6) 2.0 = Distorted circular appearance or >50% narrowing with mild to moderate PBVO 3.0 = Near disappearance of airway lumen with marked PBVO	
	Tree-in-bud (6)	1.0 = Mild and affecting <1/3 lung 2.0 = Moderate and affecting ≥1/3 to <2/3 lung 3.0 = Severe and affecting ≥2/3 lung	
Vascular Changes	PT:Ao > 1.4 (17)	3.0 if present	Highest score from either category, maximum score of 3
	PTE	1.0 = Affecting subsegmental or smaller caliber arteries 2.0 = Affecting lobar or segmental arteries in <5 lobes 3.0 = Affecting left or right MPA or lobar or segmental arteries in 5–6 lobes	

*Inspiratory CT scans are scored for attenuation and vascular changes. Airway caliber changes require comparison of inspiratory and expiratory scans. GGO, ground glass opacity; I/E: inspiratory:expiratory; MPA: main pulmonary artery; PBVO, peribronchovascular opacification; PT:Ao, pulmonary trunk-to-aorta ratio; PTE, pulmonary thromboembolism*.

## Statistical Analysis

Descriptive statistics were performed when appropriate and data were presented as mean ± standard deviation. Statistics were performed using Stata 13.1 IC Software (StataCorp LP, College Station, TX). Data were evaluated for normality using the Shapiro-Wilk normality test and were found to be normally distributed. Two-tailed *t*-tests were used to compare variables between groups stratified by P:F and again when stratified by CT disease severity score. Paired single-tailed *t*-tests were used to compare the change in P:F following BAL between groups stratified by P:F and again when stratified by CT disease severity score. Linear regression was used to assess the relationship between pre-BAL P:F and CT disease severity score. Statistical significance was defined as alpha <0.05 for single- and two-tailed *t* tests and linear regression.

## Results

### Patient Demographics

From April 2018 to August 2019, 49 dogs with RD underwent thoracic CT with BAL with or without tracheobronchoscopy. Thirty dogs were excluded due to lack of availability of primary author to facilitate study enrollment, incomplete required diagnostic tests, fulfillment of exclusion criteria, and/or the inability to obtain client consent, whether due to ethical or logistic considerations or to a subjective concern for patient instability. Nineteen dogs were initially enrolled, but one was later excluded due to inability to obtain ABG and one due to technical complications precluding ventilator-acquired pulmonary mechanics. Therefore, 17 dogs were included in the final data analysis, with mixed breed dogs (24%, 4/17), Shih Tzus (12%, 2/17), and Miniature Schnauzers (12%, 2/17) being the most common breeds represented. One each of Labrador Retriever, Boxer, Great Dane, English Pointer, American Staffordshire Terrier, Miniature Pinscher, Standard Poodle, Weimaraner, and Pembroke Welsh Corgi were also included. There were 10 (59%) neutered females and 7 (41%) neutered males. The mean ± SD age was 8 ± 4 years (range, 1–15 years old). The mean body weight was 18.8 ± 13.3 kg (range, 6.0–51.6 kg) and BCS was 5.5 ± 1.6 (range, 3.0–9.0/9.0; not recorded for 1 dog).

On admission, mean heart rate was 118 ± 26 bpm (range, 72–160 bpm), mean respiratory rate was 57 ± 36 brpm (range 16–150 brpm; recorded as “pant” for 3 dogs), and mean rectal temperature was 101.8 ± 1.3°F (range, 99.9–105.7°F). Within 1 week of diagnostic procedures, mean WBC count was 17.5 ± 11.0 × 10^3^ μl (range, 4.6–40.6 × 10^3^ μl; not recorded for 1 dog).

### Oxygen Requirements and Post-procedural Complications

SpO_2_ was not a required inclusion criterion and so was only recorded in 4/17 (24%) patients prior to GA and advanced diagnostic testing. Two were recorded while breathing room air (dog 4: 84% and dog 17: 90% in Table 2) and two while receiving ~40% supplemental oxygen (dog 10: 85% and dog 13: 85–90%). Those patients that had SpO_2_ recorded were the only patients enrolled who were provided supplemental oxygen by their attending clinician prior to diagnostic testing. This was achieved via flow-by, nasal cannulation, oxygen cage, and/or endotracheal intubation.

**Table 2:** Signalment, diagnoses, length of hospitalization, post-procedural complications, outcomes, and CT disease severity scores in 17 dogs undergoing thoracic computed tomography and bronchoalveolar lavage to evaluate lower respiratory tract disease stratified into groups by arterial partial pressure of oxygen to fractional inspired oxygen ratio.

	**Dog**	**Age (years)**	**Sex**	**Breed**	**Diagnoses**	**LOH (days)**	**Post-procedural complications**	**Outcome**	**CT group**
ABG Group 1 (P:F >300)	1	4	FS	Labrador Retriever	Suspect parasitic pneumonia (*Trichinella*)	6	None	Survived to discharge	2
	2	15	FS	Mixed Breed	TC, MSB collapse, BM, bronchiectasis/bronchiolectasis, MVD/tricuspid valve degeneration, PH, solitary lung nodule	3	None	Survived to discharge	2
	3	14	MN	Shih Tzu	Laryngeal collapse, TC, MSB, BM, bronchiolectasis, CB, solitary lung cyst	4	Mild	Survived to discharge	1
	4	10	FS	Boxer	PVOD	1	None	Euthanized	2
	5	4	FS	Great Dane	Bacterial pneumonia (*E. coli*)	1	None	Survived to discharge	1
	6	10	MN	Mixed Breed	TC, MSB, BM, MVD with LA enlargement, PH, CB	1	None	Survived to discharge	2
	7	7	MN	English Pointer	Pulmonary adenocarcinoma (intrapulmonary metastasis) with hemorrhage and necrosis, heartworms in arteries, intralymphatic tumor emboli	1	None	Survived to discharge	2
	8	2	MN	American Staffordshire Terrier	Bronchiectasis/bronchiolectasis suspected secondary to recurrent AP (latter not present at time of CT)	1	None	Survived to discharge	1
	9	13	FS	Miniature Pinscher	Bronchiectasis, bronchomalacia, CB, acute canine infectious respiratory disease complex (*Bordetella*)	1	Mild	Survived to discharge	1
ABG Group 2 (P:F ≤300)	10	9	MN	Mixed Breed	TC, MSB collapse, BM, lymphoma of sternal lymph nodes, consolidation due to either lymphoma or AP	3	None	Euthanized	2
	11	2	MN	Miniature Schnauzer	CB and bronchiolitis, necrotizing pneumonia	1	None	Survived to discharge	2
	12	10	FS	Miniature Schnauzer	Histiocytic sarcoma	3	None	Survived to discharge	1
	13	1	FS	Standard Poodle	Eosinophilic bronchopneumopathy	3	None	Died	2
	14	11	FS	Weimaraner	Pulmonary carcinoma with intrapulmonary metastasis, lung cysts, PTE	3	None	Survived to discharge	2
	15	9	FS	Shih Tzu	CB, bronchomalacia, bronchiectasis, secondary mycoplasma bronchitis	1	None	Survived to discharge	2
	16	13	MN	Mixed Breed	Dysphagia/macro-aspiration with repetitive AP; BM, bronchiectasis	1	None	Survived to discharge	2
	17	9	FS	Pembroke Welsh Corgi	PH, secondary bacterial pneumonia, TC, uncharacterized bronchiolar disease, suspect pulmonary fibrosis	4	Moderate to severe	Survived to discharge	1

*AP, aspiration pneumonia; BM, bronchomalacia; CB, chronic bronchitis; FS, female spayed; LOH, length of hospitalization; MN, male neutered; MSB, main stem bronchi; MVD, myxomatous valvular degeneration; PH, pulmonary hypertension; PTE, pulmonary thromboembolism; PVOD, pulmonary veno-occlusive disease; TC, tracheal collapse*.

The overall incidence of post-procedural complications was 3/17 (18%), with two dogs (dogs 3 and 9) having mild complications requiring short-term oxygen supplementation and one dog (dog 10) experiencing a moderate to severe post-procedural complication. Dog 10 was in respiratory failure prior to diagnostic testing and SpO_2_ following extubation was ~65% on 40–60% oxygen. After ~1 h of oxygen supplementation, the dog returned to the same clinical state and level of hypoxemia as was noted prior to GA and diagnostics.

Two additional dogs (dogs 13 and 17) were oxygen- or mechanical-ventilation-dependent prior to respiratory diagnostics but did not experience respiratory or cardiovascular decompensation induced by GA or testing. Therefore, they were not categorized as having direct post-procedural complications. Dog 13 presented in respiratory distress and required immediate intubation and mechanical ventilation prior to diagnostic procedures due to labored breathing and desaturation. Diagnostic testing was pursued shortly thereafter and the dog was then maintained on the mechanical ventilator for continued care. Directly following the procedure, the dog's SpO_2_ was 92% while receiving mechanical ventilation and it was considered hemodynamically stable. Dog 17 required oxygen supplementation before the procedure and had a post-procedural SpO_2_ of 94% on room air. The dog was maintained in an oxygen cage at ~30–40% oxygen for the following 3 days at the attending clinician's discretion and was eventually discharged following initiation of medical therapy and gradual weaning of oxygen supplementation.

### Anesthetic Protocols

Most dogs (13/17; 76.5%) received premedication with nalbuphine hydrochloride (Hospira Inc., Lake Forest IL) IV at 0.4–0.5 mg/kg ± dexmedetomidine hydrochloride (Dexdomitor®, Zoetis Services, LLC, Parsippany NJ) at 3–5 mcg/kg IV. Dog 13 received hydromorphone hydrochloride (Hospira Inc., Lake Forest IL) 0.05 mg/kg IV for premedication and rocuronium bromide (X-Gen, Big Flats NY) 0.3 mg/kg IV to facilitate imaging. Dog 9 received butorphanol (Torbugesic®, Zoetis Services, LLC, Parsippany NJ) 0.5 mg/kg IV, dog 2 received acepromazine (VetOne, Boise ID) 0.02 mg/kg and morphine sulfate (Hikma Pharmaceuticals, Eatontown NJ) 0.5 mg/kg IV, and dog 17 received dexmedetomidine 5 mcg/kg and hydromorphone 0.1 mg/kg for premedication. There were no variations from propofol induction or CRI protocols for any dog.

### Arterial Blood Gas, Ventilator-Acquired Pulmonary Mechanics, and CT Severity Score Results

Arterial catheter placement was successful in 14/17 (82%) patients and 3/17 (18%) required femoral artery puncture. The mean pre-BAL P:F was 306 ± 98 (range, 50–493) and post-BAL P:F was 267 ± 118 (range, 73–470). The mean change in P:F (defined as post minus pre-BAL P:F) was −38 ± 119 (range, −170 to +334), reflecting a decrease in P:F following BAL.

Ventilator-calculated mean specific compliance was 1.3 ± 0.6 ml/cm H_2_O/kg (range, 0.3–2.7 ml/cm H_2_O/kg) and specific resistance was 1.2 ± 1.2 cm H_2_O/L/s/kg (range, 0.1 ± 3.7 cm H_2_O/L/s/kg). The mean CT disease severity scores were 1.5 ± 0.7 in the attenuation category (reflective of parenchymal disease; range, 0.4–2.6), 2.2 ± 0.8 in airway caliber category (reflective of dynamic or static decreases in airway luminal diameter; range, 1.0–3.0), and 1.1 ± 1.5 in the vascular category (reflective of pulmonary hypertension or obstructive vascular disease; range, 0–3.0). The mean overall CT disease severity score was 4.8 ± 2.2 (range, 1.4–8.3).

### Length of Hospitalization and Mortality

Mean LOH was 0.9 ± 1.5 days (range, 0–4.0 days) with 14/17 dogs (83%) surviving to hospital discharge. Dogs 4 and 10 were not included in calculation of LOH due to euthanasia on the same day as the procedure. Euthanasia was elected by owners due to the severity of RD, limited treatment options, and poor prognosis [i.e., pulmonary venoocclusive disease (13) in dog 4, chemotherapy-refractory lymphoma in dog 10] and not due to complications associated with the procedure. There were 10/15 (67%) that had zero days of hospitalization due to being discharged from the hospital the same day as diagnostic procedures.

Of the three dogs (18%) that did not survive, the aforementioned two dogs were euthanized the same day as diagnostic testing and an additional dog died in the hospital without attempt at cardiopulmonary resuscitation; none of these were considered a direct result of the diagnostic procedures. The death (dog 13) was attributed to systemic complications of severe acute pancreatitis that developed 2 days following thoracic CT and BAL. This dog had been demonstrating marked improvement of its RD while receiving mechanical ventilation and the pancreatitis was thought to be secondary to propofol received during ventilation (18). Individual outcomes for each dog are detailed in Table 2.

### ABG Group Stratification

Based on pre-BAL P:F, nine dogs were assigned to ABG group 1 (mild hypoxemia, mean 370.1 ± 62.0) and eight dogs were assigned to ABG group 2 (moderate to severe hypoxemia, mean 234.3 ± 79.4). Mean gender (*p* = 0.79), age (*p* = 0.73), body weight (*p* = 0.39), BCS (*p* = 1.0), presenting heart rate (*p* = 0.17), respiratory rate (*p* = 0.17), temperature (*p* = 0.17), and white blood cell counts (*p* = 0.21) did not differ between groups.

Data obtained from the mechanical ventilator and thoracic CT scores were compared between ABG groups. There was no significant difference between mean specific compliance (ABG group 1, 1.4 ± 0.5 ml/cm H_2_O; ABG group 2, 1.2 ± 0.7 ml/cm H_2_O) or resistance (ABG group 1, 1.0 ± 1.2 cm H_2_O/L/s; ABG group 2, 1.4 ± 1.4 cm H_2_O/L/s) between groups (*p* = 0.50 and 0.47, respectively). Mean total CT disease severity score for ABG group 1 (4.3 ± 2.1) was not significantly different compared to ABG group 2 (5.3 ± 2.2; *p* = 0.39).

There was no significant difference between mean post-BAL P:F (ABG group 1, 298.5 ± 124.2; ABG group 2, 233.4 ± 108.9) between groups (*p* = 0.27). The mean change in P:F following BAL in ABG group 1 was −71.6 ± 139.0, which reflects a decrease in P:F following BAL. The mean post-BAL P:F in ABG group 1 was not significantly different from the pre-BAL P:F (*p* = 0.14). In ABG group 2, the mean change in P:F was −0.9 ± 85.8 and the post-BAL P:F was not significantly different from the pre-BAL P:F (*p* = 0.99). There was no significant difference in the change in P:F following BAL between ABG groups (*p* = 0.14). There was also no significant difference in the incidence of post-procedural complications (*p* = 0.92), LOH (*p* = 0.51), or mortality (*p* = 0.48) between ABG groups.

### CT Group Stratification

When patients were stratified into groups based on CT disease severity scores, six were assigned to CT group 1 (mean, 2.5 ± 0.5) and 11 to CT group 2 (mean, 6.0 ± 1.6). Mean gender (*p* = 0.65), age (*p* = 0.87), body weight (*p* = 0.85), BCS (*p* = 0.54), presenting respiratory rate (*p* = 0.83), temperature (*p* = 0.79), and white blood cell counts (*p* = 0.42) did not differ between groups. CT group 1 had a significantly higher mean presenting heart rate (136 ± 27 bpm) than CT group 2 (109 ± 21 bpm; *p* = 0.04).

Data obtained from the mechanical ventilator were compared between CT groups. There was no significant difference between mean specific compliance (CT group 1, 1.3 ± 0.5 ml/cm H_2_O; CT group 2, 1.3 ± 0.7 ml/cm H_2_O) or resistance (CT group 1, 1.0 ± 0.7 cm H_2_O/L/s; CT group 2, 1.3 ± 1.5 cm H_2_O/L/s) between groups (*p* = 0.96, 0.71, respectively).

There was no significant difference in mean pre-BAL P:F (CT group 1, 366 ± 87; CT group 2, 274 ± 91) between groups (*p* = 0.06). There was no significant difference in mean post-BAL P:F (CT group 1, 269 ± 121; CT group 2, 268 ± 123) between groups (*p* = 0.99). The mean change in P:F following BAL in CT group 1 was −97±139, which reflects a decrease in P:F following BAL and the mean post-BAL P:F in CT group 1 was not statistically significant from the pre-BAL P:F (*p* = 0.14). In CT group 2, the mean change in P:F was 6 ± 100, reflecting a minimal change in P:F following BAL. The post-BAL P:F in CT group 2 was not significantly different from the pre-BAL P:F (*p* = 0.90). There was no significant difference in the change in P:F between CT groups (*p* = 0.07). There were two incidences of mild and one incidence of moderate to severe post-procedural complications in CT group 1 and none in CT group 2. There were no significant differences in LOH (*p* = 0.78) or mortality (*p* = 0.18) between CT groups.

### Relationship Between pre-BAL P:F and Total CT Score

**Figure 2:**
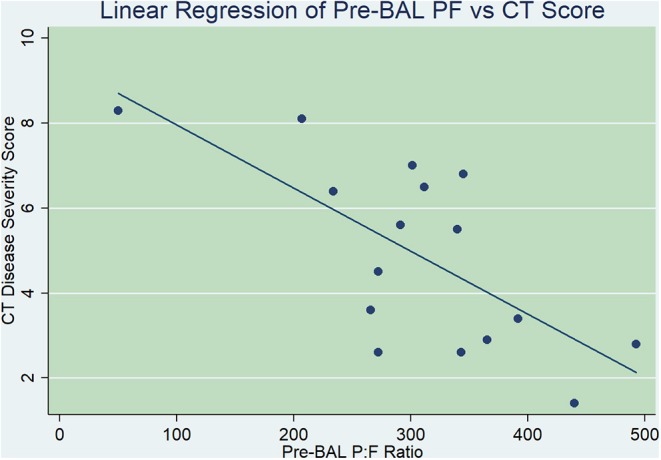
Linear regression comparing pre-bronchoalveolar lavage (BAL) arterial partial pressure of oxygen-to-fractional inspired oxygen ratio (P:F, *x*-axis) with thoracic computed tomography (CT) disease severity score (*y*-axis). There was a statistically significant linear correlation found between pre-BAL P:F and CT disease severity scores, where for each unit change in pre-BAL P:F, 43% of the variation in CT score could be predicted (*p* = 0.0042).

When evaluating the relationship between efficiency of gas exchange (pre-BAL P:F) and global anatomic measures of disease (total CT disease severity score), there was a significant linear correlation noted (*p* = 0.0042). Linear regression analysis yielded a prediction equation where CT score = 447.87–29.77104 × (pre-BAL P:F). The *R*^2^ value (0.43) indicated that for each unit change in pre-BAL P:F, 43% of the variation in CT score could be predicted (Figure 2).

## Discussion

Our study demonstrates that dogs with RD undergoing GA for thoracic CT and BAL have a low incidence of post-procedural complications with no mortality directly attributed to advanced diagnostic testing, regardless of disease severity. Additionally, ABG data demonstrated that BAL did not cause significant compromise of pulmonary gas exchange in this patient population and that P:F was moderately well correlated with a novel CT disease severity scoring system. This suggests promise for the application of this CT score in dogs with RD in providing an objective and holistic measure of disease severity without the requirement of ABG collection.

The signalment and final diagnoses of the study dogs were diverse, which reflects the population of dogs presenting for advanced respiratory diagnostics at our institution. The range in disease severity, whether measured using P:F or CT scores, was also broad and included a substantial proportion of severely affected dogs. Additionally, as listed in Table 2, dogs frequently had multiple co-morbid respiratory disorders (range, 1–7 individual RDs).

In general, dogs perceived to have severe disease may be less likely to undergo advanced diagnostic testing as they may be thought to be poor or unstable candidates for GA and BAL. One of the key findings of this study was that GA with advanced diagnostic testing could be safely performed with minimal post-procedural complications and no direct procedure-associated mortality in dogs with multiple severe respiratory disorders. However, as there is always risk of RD exacerbation associated with advanced diagnostic testing, one goal of our study was to determine if noninvasive criteria obtained with minimal stress to the patient and prior to advanced diagnostic testing could predict complications. The Systemic Inflammatory Response Syndrome (SIRS) criteria, which include temperature, pulse, respiratory rate, and WBC count, have been previously proposed as potential markers of disease severity in dogs despite a range in sensitivity and specificity from 77 to 97% (19, 20). In the current study, dogs with more severe disease, as stratified by CT scores, had a lower heart rate prior to diagnostic testing than those with less severe disease. It is suspected that the lower heart rate in this group was caused by increased vagal tone secondary to severe global RD. These results suggest that heart rate could potentially be used as an indicator of disease severity during triage of dogs in respiratory distress prior to advanced diagnostic testing, but further study is needed to better evaluate this relationship.

The overall incidence of post-procedural complications was 18% in the included patient population. Reported post-procedural complications of BAL in dogs include air-flow limitation, need for supplemental oxygen and worsening of preexisting RD or cough (1, 2), but rates of post-procedural complication directly attributed to the effect of comprehensive diagnostic procedures have not been previously evaluated. In the current study, suspected and/or confirmed hypoxemia with need for supplemental oxygen was the only complication observed. There was no statistically significant difference in the incidence of post-procedural complications in groups when stratified by ABG data. However, post-procedural complications were only seen in CT group 1 and not in CT group 2. A higher incidence of post-procedural complications would not typically be expected in a more mildly affected patient group, but due to the small patient number and low incidence of post-procedural complications overall, interpretation of low-powered statistical comparisons is limited and likely lacks biological importance. Also, direct quantification of the effect diagnostic procedures had on outcomes was challenging considering the severity of clinical signs in this patient population and limitations in differentiating between the effect of diagnostic procedures and progression of underlying disease as an explanation for any change in clinical status. There was also no standardization or requirement for objective measurement of oxygenation status prior to a clinician determining the need for oxygen supplementation before or after the procedures, so dogs in need of oxygen supplementation may not have received it and others may have been provided supplemental oxygen without requirement. An attempt was made to circumvent this effect by only counting post-procedural complications in patients with a clinically significant change in their respiratory stability following BAL, but further research is needed with standardized measurement of oxygenation status prior to and after recovery from GA to determine the true incidence of post-procedural complications in this patient population.

The anesthetic protocols used were similar for all dogs included in this study. Premedication largely included a kappa or mu agonist opioid with or without an alpha-2 agonist, followed by propofol for induction and maintenance of anesthesia. Though there were no major complications directly attributed to anesthetic medications and/or GA, both opioids and alpha-2 agonists have the potential for a detrimental effect on pulmonary gas exchange via depression of respiratory drive and increase in systemic vascular tone, respectively. GA and recumbency alone likely contributed to ventilation–perfusion mismatching by causing atelectasis, though this effect was mitigated by minimizing anesthetic time prior to diagnostics and maintaining patients in sternal recumbency. Standardization of sedation and anesthetic protocols may have helped control for the contribution to ventilation and perfusion mismatching by anesthetic protocols that were otherwise attributed to BAL. Due to small sample size and variation in anesthetic protocols, the effects of GA were not directly evaluated in the current study.

There was a minimal decrease in P:F following BAL when evaluating all dogs together. However, there was a large range and standard deviation noted that represented both improvement and decline in P:F following BAL. There are several possible explanations for this variation in the sensitivity of pulmonary function to BAL, including the effects of GA, anesthetic technique, endotracheal intubation, and underlying disease etiology. Only the effect of disease severity (quantified by ABG data and CT disease severity scores) was evaluated as an explanation for this wide variation in response to BAL and is discussed below. Further research with inclusion of a control population, standardization of anesthetic technique and a larger patient population would be required to further investigate this relationship. Inclusion of ABG collection prior to and after GA would also aid in differentiation of the effects of GA from the potential effects of BAL on pulmonary function.

The mean LOH in the included patient population was <1 day, primarily due to the majority of included patients (67%) being able to undergo advanced diagnostic testing as an outpatient procedure. Though all-cause mortality was 18%, no deaths or euthanasias were attributed to direct complications of diagnostic testing. Although the 18% reported in this study is higher than the 2% mortality previously reported (3), it is possible that this discrepancy was due to the current study including a more severely affected patient population under GA for longer periods of time. A direct comparison cannot be made as disease severity was not evaluated in the past study.

Euthanasia contributed to the higher mortality rate reported in the current study but was also not a direct result of decompensation from advanced diagnostics. The decision to euthanize the two dogs included was made due to the severity of clinical signs and improbability of seeing clinical improvement with therapy, rather than a clinical decline observed following diagnostic procedures. One dog was suspected to have PVOD antemortem (later confirmed via necropsy), which has a reported 100% mortality rate and no viable treatment options (13). The other had lymphoma that was progressing despite chemotherapeutics and was in severe respiratory failure. The link between advanced diagnostics and mortality in this study is analogous to taking thoracic radiographs in a population of dogs with neoplasia being screened for metastatic disease. While radiography may identify pulmonary metastases and lead to a decision of euthanasia, radiography is not the direct cause of mortality. Perhaps a more accurate interpretation of mortality in the current study is that euthanasia and death directly associated with diagnostic testing were 0%; however, when euthanasia indirectly associated with advanced diagnostics testing and death due to unrelated disease were considered, mortality was 18%.

The relationship between increasing disease severity and outcomes in dogs undergoing GA with advanced diagnostic testing for RD had not been previously evaluated. When stratified using ABG values, there was no statistically significant difference between groups in signalment, body weight/BCS, minimally invasive SIRS markers, specific compliance and resistance, CT scores, post-procedural complications, LOH, mortality, post-BAL P:F, or the change in P:F following BAL. When stratified using CT scores, there was no statistically significant difference between groups in signalment, body weight/BCS, minimally invasive SIRS markers except for HR, specific compliance and resistance, LOH, mortality, pre- and post-BAL P:F, or the change in P:F following BAL. However, regardless of disease stratification, it was interesting to note that there was a greater decrease in P:F following BAL in the mildly affected groups and minimal change in P:F in moderate to severely affected groups. Though this difference should not be over-interpreted in the absence of statistical significance, it is possible that dogs with mild disease may be more sensitive to the effect of BAL on pulmonary gas exchange than those more severely affected. A larger sample size would be necessary to further investigate this relationship.

To better characterize global respiratory anatomic changes relating to the pulmonary parenchyma, airways and vasculature, a novel thoracic CT disease severity scoring system was introduced and compared to ABG data in the current study. There was a significant relationship between these two variables, suggesting that with increasing disease severity, a decreasing P:F correlates with an increasing CT score. Though further study is needed prior to clinical application, there is great potential in using this comprehensive thoracic CT scoring system to allow objective global disease severity quantification prior to BAL in this patient population without the need for ABG collection.

## Limitations and Conclusions

There were several additional limitations to the current study. First, the lack of a control group limits interpretation of ABG data. For example, even though a minimal change in P:F ratio was seen in dogs with moderate to severe disease, it is unclear how much of the change in P:F was due to BAL alone, rather than a result of preexisting RD. Second, the sample size was relatively small. Observed variation in signalment and underlying co-morbid disease in the study dogs, while representative of our actual patient population, provides challenges when considering specific disease(s). A larger sample size may help to both increase statistical power and facilitate evaluation of subgroups based on definitive diagnosis. Finally, a selection bias regarding the enrollment of dogs in the study is possible. Though a wide variety of disease etiologies and severity were represented, it is possible that owners of dogs who were perceived to have more severe disease declined diagnostic testing or study enrollment due to concern for complications.

Dogs in this study with RD undergoing GA with advanced diagnostics, including thoracic CT and BAL, had a low incidence of post-procedural complications and no mortality directly associated with diagnostic testing, regardless of disease severity. Thus, results support that these diagnostic tests can be performed safely even in dogs with severe disease, allowing more precise and comprehensive diagnosis to optimize appropriate therapy and improve prognostication. A novel thoracic CT disease severity scoring system correlated well with ABG data, supporting that global anatomic changes in the pulmonary parenchyma, airways, and vasculature correlate with efficiency of gas exchange. Additional studies are needed to validate this scoring system and to further elucidate the relationships between specific RDs and outcomes in dogs undergoing advanced diagnostic testing.

## Data Availability Statement

The raw data supporting the conclusions of this article will be made available by the authors, without undue reservation, to any qualified researcher.

## Ethics Statement

The study protocol was approved by the University of Missouri Animal Care and Use Committee (IACUC #9286). Informed client consent was obtained prior to study enrollment.

## Author Contributions

ZB, AB, and CRein designed the study. ZB wrote the manuscript. ZB and LS compiled and analyzed the data. IM and CRein developed the CT disease severity scoring system and IM provided the CT disease severity scores. Results were interpreted and the manuscript was critically revised by ZB, AB, IM, CReic, and CRein. All authors read and approved the final manuscript.

## Conflict of Interest

The authors declare that the research was conducted in the absence of any commercial or financial relationships that could be construed as a potential conflict of interest.

## Abbreviations

ABG: arterial blood gas
ARDS: acute respiratory distress syndrome
BAL: bronchoalveolar lavage
BCS: body condition score
CT: computed tomography
EtCO_2_: end-tidal carbon dioxide
F_I_O_2_: fractional inspired oxygen
GA: general anesthesia
LOH: length of hospitalization
P:F: arterial oxygen partial pressure to fractional inspired oxygen ratio
PEEP: positive end-expiratory pressure
PPV: positive pressure ventilation
RD: respiratory disease
SIRS: systemic inflammatory response syndrome
SpO_2_: peripheral capillary oxygen saturation.

## References

[1] Finke MD. Transtracheal wash and bronchoalveolar lavage. Top Companion Anim Med. (2013) 28:97–102. 10.1053/j.tcam.2013.06.003 24182997

[2] Cooper ES Schober KE Drost WT. Severe bronchoconstriction after bronchoalveolar lavage in a dog with eosinophilic airway disease. J Am Vet Med Assoc. (2005) 227:1257–62. 10.2460/javma.2005.227.1257 16266013

[3] Hawkins EC DeNicola DB Plier ML. Cytological analysis of bronchoalveolar lavage fluid in the diagnosis of spontaneous respiratory tract disease in dogs: a retrospective study. J Vet Intern Med. (1995) 9:386–92. 10.1111/j.1939-1676.1995.tb03298.x 8558485

[4] Schnabel RM van der Velden K Osinski A Rohde G Roekaerts PM Bergmans DC. Clinical course and complications following diagnostic bronchoalveolar lavage in critically ill mechanically ventilated patients. BMC Pulm Med. (2015) 15:107. 10.1186/s12890-015-0104-1 26420333 PMC4588466

[5] Wilkins PA Otto CM Baumgardner JE Dunkel B Bedenice D Paradis MR Acute lung injury and acute respiratory distress syndromes in veterinary medicine: consensus definitions: the dorothy russell havemeyer working group on ALI and ARDS in veterinary medicine. J Vet Emerg Crit Care. (2007) 17:333–9. 10.1111/j.1476-4431.2007.00238.x

[6] Masseau I Reinero CR. Thoracic computed tomographic interpretation for clinicians to aid in the diagnosis of dogs and cats with respiratory disease. Vet J. (2019) 253:105388. 10.1016/j.tvjl.2019.105388 31685132

[7] Harmanci E Kebapci M Metintas M Ozkan R. High-resolution computed tomography findings are correlated with disease severity in asthma. Respiration. (2002) 69:420–6. 10.1159/000064018 12232449

[8] Wells AU Desai SR Rubens MB Goh NS Cramer D Nicholson AG. Idiopathic pulmonary fibrosis: a composite physiologic index derived from disease extent observed by computed tomography. Am J Respir Crit Care Med. (2003) 167:962–9. 10.1164/rccm.2111053 12663338

[9] Mitsunobu F Mifune T Ashida K Hosaki Y Tsugeno H Okamoto M. Influence of age and disease severity on high resolution CT lung densitometry in asthma. Thorax. (2001) 56:851–6. 10.1136/thorax.56.11.851 11641509 PMC1745946

[10] Sanders DB Li Z Brody AS Farrell PM. Chest computed tomography scores of severity are associated with future lung disease progression in children with cystic fibrosis. Am J Respir Crit Care Med. (2011) 184:816–21. 10.1164/rccm.201105-0816OC 21737586 PMC3208650

[11] Muller NL Mawson JB Mathieson JR Abboud R Ostrow DN Champion P. Sarcoidosis: correlation of extent of disease at CT with clinical, functional, and radiographic findings. Radiology. (1989) 171:613–8. 10.1148/radiology.171.3.2717730 2717730

[12] Coia ME Hammond G Chan D Drees R Walker D Murtagh K. Retrospective evaluation of thoracic computed tomography findings in dogs naturally infected by Angiostrongylus vasorum. Vet Radiol Ultrasound. (2017) 58:524–34. 10.1111/vru.12505 28429379

[13] Reinero CR Jutkowitz LA Nelson N Masseau I Jennings S Williams K. Clinical features of canine pulmonary veno-occlusive disease and pulmonary capillary hemangiomatosis. J Vet Intern Med. (2019) 33:114–23. 10.1111/jvim.15351 30499214 PMC6335444

[14] Jaffey JA Harmon M Masseau I Williams KJ Reinero C. Presumptive development of fibrotic lung disease from Bordetella bronchiseptica and post-infectious bobliterronchiolitis obliterans in a dog. Front Vet Sci. (2019) 6:352. 10.3389/fvets.2019.00352 31649945 PMC6795681

[15] Bradbrook CA Clark L Dugdale AH Burford J Mosing M. Measurement of respiratory system compliance and respiratory system resistance in healthy dogs undergoing general anaesthesia for elective orthopaedic procedures. Vet Anaesth Analg. (2013) 40:382–9. 10.1111/j.1467-2995.2012.00778.x 23433215

[16] Jaffey JA Wiggen K Leach SB Masseau I Girens RE Reinero CR. Pulmonary hypertension secondary to respiratory disease and/or hypoxia in dogs: clinical features, diagnostic testing and survival. Vet J. (2019) 251:105347. 10.1016/j.tvjl.2019.105347 31492386

[17] Sutherland-Smith J Hankin EJ Cunningham SM Sato AF Barton BA. Comparison of a computed tomographic pulmonary trunk to aorta diameter ratio with echocardiographic indices of pulmonary hypertension in dogs. Vet Radiol Ultrasound. (2018) 59:18–26. 10.1111/vru.12540 28857335

[18] Devlin JW Lau AK Tanios MA. Propofol-associated hypertriglyceridemia and pancreatitis in the intensive care unit: an analysis of frequency and risk factors. Pharmacotherapy. (2005) 25:1348–52. 10.1592/phco.2005.25.10.1348 16185179

[19] Hauptman JG Walshaw R Olivier NB. Evaluation of the sensitivity and specificity of diagnostic criteria for sepsis in dogs. Vet Surg. (1997) 26:393–7. 10.1111/j.1532-950X.1997.tb01699.x 9381665

[20] Silverstein D Systemic inflammation response syndrome & sepsis. Part 1: recognition & diagnosis. Todays Vet Pract. (2015) 38–44. Available online at: https://todaysveterinarypractice.com/systemic-inflammatory-response-syndrome-sepsis-part-1-recognition-diagnosis/

